# Loss of miR-133a expression associated with poor survival of breast cancer and restoration of miR-133a expression inhibited breast cancer cell growth and invasion

**DOI:** 10.1186/1471-2407-12-51

**Published:** 2012-02-01

**Authors:** Zheng-sheng Wu, Chao-qun Wang, Ru Xiang, Xue Liu, Shan Ye, Xue-qing Yang, Gui-hong Zhang, Xiao-chun Xu, Tao Zhu, Qiang Wu

**Affiliations:** 1 Department of Pathology, Anhui Medical University, Hefei, Anhui, People's Republic of China; 2 Department of Pathology, Shanghai Medical College, Fudan University, Shanghai, People's Republic of China; 3 Department of Pathology, Dongyang People's Hospital, Dongyang, Zhejiang, People's Republic of China; 4 Department of Clinical Cancer Prevention, The University of Texas MD Anderson Cancer Center, Houston, Texas; 5 School of Life Science, University of Science and Technology of China, Hefei, Anhui, People's Republic of China; 6 Department of Pathology, Anhui Medical University, 81 Meishan Road, Hefei 230032, China

**Keywords:** Breast cancer, miRNA, miR-133a, Tumor cell invasion, Fascin1, Prognosis

## Abstract

**Background:**

miRNAs, endogenous oligonucleotide RNAs, play an important role in mammary gland carcinogenesis and tumor progression. Detection of their expression and investigation of their functions could lead to discovery of novel biomarkers for breast cancer.

**Methods:**

*In situ *hybridization was used to detect miR-133a expression in formalin-fixed paraffin-embedded breast surgical specimens from 26 benign, 34 pericancerously normal and 90 cancerous tissues. qRT-PCR was performed to assess miR-133a levels in 6 breast cell lines and 10 benign and 18 cancerous fresh breast tissue specimens. Cell viability, migration, and invasion assays were used to determine the role of miR-133a in regulation of breast cancer cell growth, migration, and invasion, respectively. Luciferase assay was performed to assess miR-133a binding to FSCN1 gene.

**Results:**

Expression of miR-133a was reduced from normal through benign to cancerous breast tissues. Expression of miR-133a was also low in breast cancer cell lines. The reduced miR-133a expression was associated with lymph nodes metastasis, high clinical stages, and shorter relapse-free survivals of patients with breast cancer. Furthermore, transfection of miR-133a oligonucleotides slightly inhibited growth but significantly decreased migration and invasion capacity of breast cancer cells, compared with negative controls, whereas knockdown of miR-133a expression induced breast cancer cell migration and invasion. In addition, we identified a putative miR-133a binding site in the 3'-untranslated region (UTR) of Fascin1 (FSCN1) gene using an online bioinformatical tool. We found that miR-133a transfection significantly reduced expression of FSCN1 mRNA and protein. The luciferase reporter assay confirmed that FSCN1 was the direct target gene of miR-133a.

**Conclusions:**

miR-133a expression was lost in breast cancer tissues, loss of which was associated with lymph nodes metastasis, high clinical stages and shorter relapse-free survivals of patients with breast cancer. Functionally, miR-133a can suppress tumor cell invasion and migration and targeted the expression of FSCN1. Future study will verify whether detection of miR-133a expression can served as a novel biomarker for breast cancer progression and patient prognosis.

## Background

MicroRNAs (miRNAs) are a class of small, non-coding RNAs of 18-24 nucleotides in length and function as negative regulators of gene expression through inhibition of target mRNA translation or degradation of target mRNA [1]. Through silence of their targeting mRNAs, miRNAs play key roles in a wide variety of biological processes, including control of embryo development [2], cell growth [3], differentiation [4] and apoptosis [5-7]. Thus, aberrant expression of miRNAs has been implicated in human carcinogenesis and cancer progression [8-12], indicating that some miRNAs can function as tumor suppressor genes or oncogenes. For example, up-regulation of several miRNAs in breast cancer cells (such as miR-9 and miR-10b) could increase tumor cell invasion and metastasis [13,14], whereas expression of miR-335 and miR-31 could inhibit breast cancer cell invasion and metastasis [15,16].

Breast cancer is the most frequently diagnosed cancer and the leading cause of cancer death for females worldwide and in the United States, accounting for 23% of the total cancer cases and 14% of the cancer deaths in the world [17]. Tumor invasion and metastasis are responsible for most of cancer-related mortality [18]. However, the mechanisms by which the factors mediate tumor metastasis are still incompletely understood, though one of these requirements is known to be the enhanced cell motility. Previous studies demonstrated that certain miRNAs (such as miR-340) were able to alter tumor cell motility [10] through regulation of c-Met gene expressions. Indeed, multiple cellular and extracellular factors can regulate cell motility by changing the actin expression or activity. In this regards, FSCN1, an actin-bundling protein, is one of the key molecules in diverse forms of actin-based motility-structures [19]. FSCN1 is normally expressed in mesenchymal, neuronal, and endothelial cells but usually not expressed in normal epithelia cells [20,21]. However, previous studies showed that FSCN1 is highly expressed in different cancer tissues or cells, such as cancers of as the mammary glands [22], colon [23], stomach [24], ovary [25], esophagus [26], and lung [27]. In our previous study, we screened expression of different miRNAs in archived formalin-fixed and paraffin-embedded breast tissue specimens by in situ hybridization [10,28], we found significant downregulated miR-133a expression in breast cancer compared to benign breast disease and miR-133a downregulation was associated with disease progression. Therefore, in this study, we first confirmed the expression of miR-133a in fresh breast tissue specimens and breast cell lines by using qRT-PCR. After that, we assessed the role of miR-133a in breast cancer cell lines and then linked miR-133a expression with FSCN1 alteration.

## Methods

### Breast tissue specimens

There are two cohorts of clinical specimens included in current study. For *in situ *hybridization assay, the tissue specimens were obtained from 90 female patients with breast cancer and 26 female patients with breast benign diseases while these patients underwent surgical treatment at the First Affiliated Hospital of Anhui Medical University from March 31 2001 to April 1 2002. For qRT-PCR assay, 18 breast cancer and 10 breast benign diseases specimens were collected from our department of Pathology between January to May 2010. These tissue specimens were placed in a cryovial, snap-frozen and stored in liquid nitrogen immediately after operation until use. The pathohistological diagnosis of the patients was according to breast tumor classification criteria of World Health Organization [29]. Histology grade was based on the Scarff-Bloom-Richardson system [30]. The median follow-up time for the breast cancer patients was 60 months and ranged from 8 to 64 months. The protocol for use of patient samples in this study was approved by our institutional review board and the informed consent form was signed by each patient or their guardians.

### Construction of tissue microarray

The formalin-fixed and paraffin-embedded tissue blocks were retrieved from the archives of the Department of Pathology, the First Affiliated Hospital of Anhui Medical University, Hefei, China. The hematoxylin and eosin-stained tissue sections were reviewed by 2 pathologists to identify the representative regions for tissue microarray construction. After that, the area of interest in the donor blocks was cored thrice with a 1 mm diameter cylinder using a tissue microarrayer (Hengtai Instruments, Liaoning, China), and transferred to the recipient paraffin block. A total of three tissue microarray blocks were constructed and sectioned for in situ hybridization analysis of miRNA expression.

### In situ hybridization

*In situ *hybridization to detect miR-133a expression in breast carcinoma and benign disease tissues was performed as described previously [10,28]. Briefly, tissue microarray sections with 3 μm in thickness were deparaffinized, rehydrated, and digested and then refixed in 4% paraformaldehyde. After that, the sections were prehybridized with 150 μl of hybridization solution and then incubated with a digoxigenin-labeled LNA probe (Exiqon, Copenhagen, Denmark) for miR-133a, U6 (positive control), and scrambled RNA (negative control) at 68°C for 20 h. In the next day, the sections were washed with 2× SSC, 1× SSC and 0.5× SSC and then incubated with a mouse antidigoxin antibody followed by streptavidin-biotin-peroxidase complex. For visualizing the positive signal, the sections were incubated in 3,3'-diaminobenzidine solution and counterstained with hematoxylin. The expression of miR-133a was reviewed and scored independently by 2 pathologists. The staining was scored as negative, if ≤ 10% of epithelial cells stained positively, whereas the staining was scored positively if > 10% of epithelial cells stained positively [31].

### Cell lines and culture

Human breast cancer ZR75-1, SKBR3, T47D, MCF-7 and MDA-MB-231 cell lines and the nontumourigenic HMEC cell line were obtained from the American Type Culture Collection (Rockville, MD). ZR75-1, SKBR3 and T47D cells were grown in Dulbecco modified Eagle medium (DMEM, Invitrogen, Carlsbad, CA), MCF-7 cells were cultured in RPMI-1640 (Invitrogen), MDA-MB-231 cells were cultured in Leibovitz's L-15 (Invitrogen) and HMEC cells were grown in Medium 171 with mammary epithelial growth supplement (Cascade Biologics) supplemented with 10% fetal bovine serum (Hyclone, Logan, UT) and incubated at 37°C in a humidified incubator containing 5% CO_2_.

### Quantitative reverse transcription polymerase chain reaction (qRT-PCR)

To detect miR-133a expression, RNA from cell lines and fresh tissue samples was extracted using the mirVana miRNA Isolation Kit (Ambion, Austin, TX) according to the manufacturer's instructions and then reversely transcribed into cDNA using SuperScript III reverse transcriptase (Invitrogen). After that, qPCR was performed using TaqMan MicroRNA Assay kits (Applied Biosystems, Foster City, CA) according to the manufacturer's instructions with a Stratagene M × 3000P Real Time PCR machine (Agilent Technologies). The PCR amplification consisted of 40 cycles (95°C for 5 s, 60°C for 20 s) after an initial denaturation at 95°C for 10 s. U6 small nuclear RNA was used as an internal control. The threshold cycle (Ct) is defined as the fractional cycle number at which the fluorescence passes the fixed threshold. The fold change of miRNA expression was calculated using the 2^-ΔΔCt ^method after normalization to U6 expression. All experiments were performed in triplicate. In addition, relative expression levels of FSCN1 mRNA were assessed by using SYBR Premix Ex Taq (Perfect Real Time) kit (TaKaRa, Dalian, China) and normalized to GAPDH mRNA. The primers for FSCN1 were 5'-CTCATCAACCGCCCCATCAT-3' (forward) and 5'-CTGCCCACCGTCCAGTATTT-3' (reverse) and GAPDH primers were 5'-TGCACCACCAACTGCTTAGC-3' (forward) and 5'-GGCATGGACTGTGGTCATGAG-3' (reverse).

### Transient miRNA and siRNA transfection

MCF-7 and MDA-MB-231 cells were selected for miR-133a transfection. Briefly, the cells were grown overnight and then transfected with either miR-133a mimic (GenePharma, Shanghai, China), 2'-O methylated single-stranded miR-133a antisense oligonucleotide (ASO, GenePharma), or negative control miRNA (GenePharma) using Lipofectamine 2000 (Invitrogen) according to the manufacturer's protocol. The miR-133a mimic contained synthetic small duplex sequences of miR-133a RNA that was able to be bioprocessed into the mature miR-133a in the cells. The sequences of negative control and ASO negative control (GenePharma) were nonhomologous to any human genome sequences, and used to eliminate the potential nonsequence-specific effects. The sequences of miR-133a mimic were: 5'-UUUGGUCCCCUUCAACCAGCUG-3' (sense), 5'-GCUGGUUGAAGGGGACCAAAUU-3' (antisense); miR-133a ASO, 5'-CAGCUGGUUGAAGGGGACCAAA-3'; NC, 5'-UUCUCCGAACGUGUCACGUTT-3' (sense) and 5'-ACGUGACACGUUCGGAGAATT-3' (antisense); ASO NC, 5'-CAGUACUUUUGUGUAGUACAA-3'. In addition, FSCN1 siRNA or control scrambled siRNA (both from Qiagen, Valencia, CA) was transfected into MCF-7 cells that were transfected with or without miR-133a ASO transfection. The target sequence of FSCN1 siRNA was AGCCCTGGGCGTGTAGTGTAA. The efficiency of RNA transfection was confirmed by real-time PCR analysis.

### Cell proliferation assay

Thirty-six hours after transient transfection, MCF-7 were harvested and sub-cultured in 96-well plates for up to 96 h. After that, cell proliferation was assessed using the CellTiter 96 AQ_ueous _MTS assay (Promega, Madison, WI) according to the manufacturer's instructions. Briefly, the MTS reagent (20 μl) was added to each well and incubated at 37°C for 2 h. Then, the absorbance at 492 nm was measured by using a microtiter plate reader (Bio-Rad, CA). The experiments were in triplicate and repeated thrice. The data were summarized as mean ± SD.

### Wound healing assay

MCF-7 cells were seeded into 6-well plates and transfected with either miR-133a ASO or ASO NC, and then the cells were allowed to grow until 100% confluency. Next, the cell layer was gently scratched through the central axis using a sterile plastic tip and loose cells were washed away. The wound healing was observed and photographed at three preselected time points (0, 24, and 48 h) in three random selected microscopic fields for each condition and time point. Sextuple assays were performed for each experiment and repeated once.

### Cell migration and invasion assay

Tumor cell migration and invasion were carried out using a Transwell insert (8 μm, Corning, NY). MCF-7 and MDA-MB-231 cells were first transfected with miR-133a mimic or ASO or control RNA. 48 h later, the cells were starved in a medium without fetal bovine serum overnight, and then 1 × 10^5 ^cells resuspended in 0.1 ml serum-free medium were added to the upper chamber and RPMI-1640 containing 20% fetal bovine serum was added to the lower chamber as a chemoattractant. For the invasion assay, the inserts were pre-coated with extracellular Matrigel (BD Biosciences, Bedford, MA). To measure the effect of miR-133a mimic on MDA-MB-231 invasion and migration potential, the cells in the upper chamber were cultured for 28 h, while 20 h incubation for miR-133a ASO on MCF-7 cells. Invaded or migrated cells were fixed and stained with 0.1% crystal violet. Five low-magnification areas (× 100) were randomly selected and counted for the cell numbers. All experiments were performed in triplicate.

### Western blot analysis

Seventy-two hours after gene transfection, the cells were washed twice with cold PBS and total cellular protein was extracted using a modified radioimmunoprecipitation assay (RIPA) lysis buffer (50 mM Tris-HCl, pH 7.4; 1% NP-40; 0.25% Na-deoxycholate; 150 mM NaCl; 1 mM EDTA; 1 mM PMSF; Aprotinin, leupeptin, pepstatin: 1 μg/ml each; 1 mM Na_3_VO_4_; 1 mM NaF). The protein concentration was then determined by a protein assay kit (Bio-Rad) and equal amounts of protein lysates (50 μg) were separated by 10% sodium dodecyl sulfate-polyacrylamide gel electrophoresis (SDS-PAGE) and then electrotransferred to the nitrocellulose membrane (GE Healthcare, Arlington Heights, IL). For Western blotting, the membranes were blocked with 5% defatted milk and incubated with primary antibodies at 4°C overnight. In the next day, the membranes were washed with PBS and then incubated with peroxidase-conjugated secondary antibodies (Santa Cruz Biotechnology, Santa Cruz, CA). The protein bands were developed with chemiluminescence (ECL) reagents (Pierce). The antibodies were anti-FSCN1 (diluted at 1:1000; Santa Cruz Biotechnologies), anti-beta-actin (diluted at 1:1000; Santa Cruz Biotechnologies).

### Luciferase reporter assay

A dual-luciferase reporter vector psiCHECK2 (Promega) was used to generate luciferase reporter constructs. The 3'-untranslated region (UTR) of human FSCN1 was amplified from human genomic DNA with primers of 5'-ATGATTCTCGAGCCTCGCTCTGGGAGTACTAGGG-3' (sense) and 5'-TATATATGCGGCCGCTGGGGCTGCAGACTGAGTTATT-3' (antisense), and inserted into the XhoI-NotI restriction sites in the 3'-UTR of the hRluc gene in psiCHECK2 vector. MCF-7 cells were grown in 24-well plates and cotransfected with 0.2 μg of psiCHECK2-FSCN1 3'UTR or psiCHECK2 control vector and 20 pmol miR-133a mimic or its negative control (GenePharma) by using Lipofectamine 2000. Each transfection was performed in triplicate and Luciferase activity was assessed 48 h after transfection using the Dual-Luciferase reporter assay system (Promega). Firefly luciferase activity was normalized to Renilla luciferase activity accordingly.

### Bioinformatical and statistical analyses

We performed bioinformatical analysis using the miRNA database TargetScan [32] (release 5.1, http://www.targetscan.org) to predict miR-133a target genes. Data were shown as means ± standard deviation (SD). All statistical analyses were performed using the SPSS 13.0 software. Differences/correlations between groups were compared using Pearson chi-square test for qualitative variables and Student t test for continuous variables. Patient relapse-free survival (RFS) and overall survival (OS) rates was analyzed using the Kaplan-Meier method and compared by log-rank analysis. *P*-value < 0.05 was considered statistically significant.

## Results

### Expression of miR-133a in breast tissue specimens and breast cell lines

**Figure 1:**
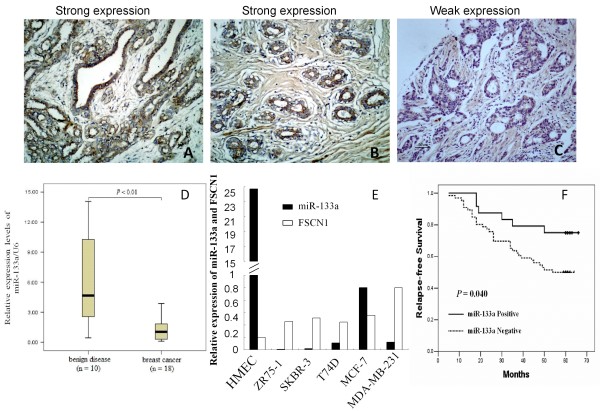
**Expression of miR-133a in breast tissue specimens and cell lines**. *A*. Strongly positive expression of miR-133a in tissues of breast benign disease. Magnification: ×200. *B*. Strongly positive expression of miR-133a in an adjacent normal breast tissue. Magnification: ×200. *C*. Weakly positive expression of miR-133a in an invasive ductal carcinoma. Magnification: ×200. *D*. Fresh tissue samples from patients with breast cancer or benign breast diseases were obtained from the surgery room. Total miRNA was extracted from the tissues and subjected to qRT-PCR analysis. *E*. Expression of miR-133a and FSCN1 in breast cancer cell lines: Breast cell lines were grown in monolayer for 3-5 days, and miRNA and total RNA was extracted and subjected to qRT-PCR analyses. *F*. Association of miR-133a expression with relapse-free survival of the patients with breast cancer.

In this study, we first assessed expression levels of miR-133a in 34 normal, 26 benign, and 90 cancerous tissue samples using in situ hybridization. Our data showed that miR-133a was predominantly expressed in luminal epithelial cells in both ductal and lobular structures in benign and normal breast specimens. In contrast, miR-133a expression was significantly lower in breast cancer tissues than in benign and normal tissues (*P *< 0.05; Figure 1 and Table 1). We further verified miR-133a expression in fresh breast tissue specimens using qRT-PCR and the data confirmed our in situ hybridization data, i.e., miR-133a expression level was significantly lower in breast cancer compared with benign breast disease (*P *< 0.01; Figure 1D). Furthermore, we chose one normal human mammary epithelial cell line HMEC and 5 breast cancer cell lines (ZR75-1, SKBR3, T47D, MCF-7 and MDA-MB-231) to analyze the expression of miR-133a using qRT-PCR. We found that miR-133a expression was significantly lower in breast cancer cell lines than that of the normal HMEC cells (Figure 1E).

**Table 1:** Expression of miR-133a in adjacent normal, benign, and cancerous breast tissues

		miR-133a expression, n (%)	
		
	**No**.	Negative	Positive
Breast cancer	90	66 (73.3)	24 (26.7)*

Benign breast disease	26	12 (46.2)	14 (53.8)

Adjacent normal	34	19 (55.9)	15 (44.1)

*P < 0.05

After that, we associated miR-133a expression with the corresponding clinicopathological data from the breast cancer patients. We observed that loss expression of miR-133a was correlated to tumor lymph nodes metastasis (*P *= 0.023) and high clinical stages (*P *= 0.021). However, there was no association between miR-133a expression and patient's age, tumor size, tumor grade, c-erbB-2 overexpression, or estrogen receptor and progesterone receptor status (Table 2).

**Table 2:** Association of miR-133a expression with clinicopathological parameters from breast cancer patients

Parameter	**No**.	MiR-133a expression, n (%)	*P-value*
Age (years)			
≤ 35	6	0 (0)	0.162
35-55	48	16 (33.3)	
> 55	36	8 (22.2)	
Tumor size (cm)			
≤ 2	4	2 (20.0)	0.523
2-5	68	18 (26.5)	
> 5	18	4 (22.2)	
Lymph node metastases			
0	29	13 (44.8)	0.023
1-3	33	7(21.2)	
> 3	28	4 (14.3)	
Tumor grade			
I	4	1 (25.0)	0.153
II	57	19 (33.3)	
III	29	4 (13.8)	
Tumor stage			
I	0	0 (0)	0.021
II	50	19(38.0)	
III	36	4 (11.1)	
IV	4	1(25.0)	
Estrogen receptor			
-	57	15 (26.3)	0.921
+	33	9 (27.3)	
Progesterone receptor			
-	56	13 (23.2)	0.705
+	34	11 (32.4)	
c-erbB-2 expression			
Low	62	14 (22.6)	0.192
High	28	10(35.7)	

### Association of miR-133a expression with survival of breast cancer patients

Next, we associated miR-133a expression with overall survival (OS) and relapse-free survival (RFS) of breast cancer patients using Kaplan-Meier survival analyses. As shown in Figure 1F, patients whose primary tumors did not express miR-133a (n = 66) had a mean RFS of 45.5 months (at a 50% 5-year RFS rate), whereas patients with tumors expressing miR-133a (n = 24) had a mean RFS time of 56.6 months (at a 75% 5-year RFS rate, *P *= 0.040). However, no unequivocal significant association of miR-133a with OS was found (*P *= 0.059, data not shown). Among the patients without tumor lymph node metatastasis, the mean RFS miR-133a expression was higher than that of patients without miR-133a expression, but it did not reach statistical significance (92.3% and 81.3%, *P *> 0.05).

### Effect of miR-133a on breast cancer cell growth

**Figure 2:**
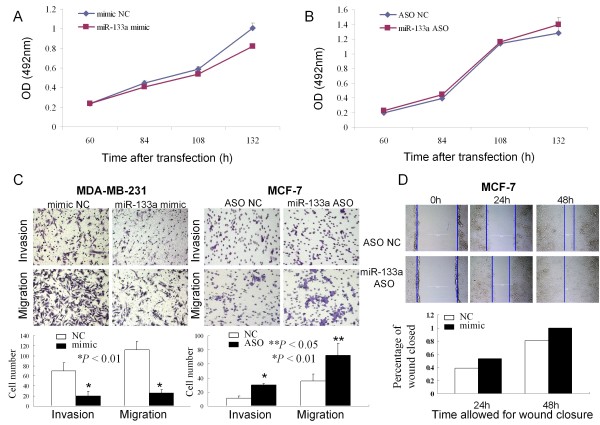
**Regulation of breast cancer cell growth, invasion and migration by miR-133a**. *A*. MCF-7 cells were grown and transiently transfected with miR-133a mimic or negative control, and cell proliferation was then assessed afterwards. The experiments were performed in triplicate and repeated thrice. *B*. MCF-7 cells were grown and transiently transfected with miR-133a ASO or negative control, and cell proliferation was assessed afterwards. The experiments were performed in triplicate and repeated thrice. *C*. Transwell migration and Matrigel invasion assays. MDA-MB-231 and MCF-7 cells were grown and transiently transfected with miR-133a mimic or miR-133a ASO for 2 days and subjected to migration and invasion assays. Representative photographs (upper) and quantification (lower) are shown. Magnification: × 200. *D*. Wound healing assay. Cells were transfected with ASO NC or miR-133a ASO for 72 h. Images were taken at 0, 24, and 48 h. Quantification of cell motility by measuring the distance between the invading front of cells in three random selected microscopic fields for each condition and time point. The degree of motility is expressed as percent of wound closure as compared with the zero time point. Magnification: × 100. * *P *< 0.05; ** *P *< 0.01.

Next, we chose MCF-7 cell line as model for the following gain-of-function and loss-of-function analysis. To evaluate the effect on cell proliferation, MCF-7 cells were transiently transfection of miR-133a mimic or miR-133a ASO. Compared with NC group, miR-133a mimic-transfected MCF-7 cells showed a time-dependent reduction of cell proliferation, i.e., rates of growth inhibition were 0.8%, 8.3%, 8.8% and 18.7% for 60 h, 84 h, 108 h, and 132 h after gene transfection, respectively (*P *< 0.05, Figure 2A). In contrast, cells transfected with miR-133a ASO revealed an increased trend of growth rates compared to the ASO NC transfection, although the difference did not reach significance (*P *> 0.05, Figure 2B).

### Effects of miR-133a on breast cancer cell migration and invasion

The potential impact of miR-133a on cell migration and invasion were assessed using transwell migration and invasion assays. After MDA-MB-231 and MCF-7 cells were selected for expression and knockdown of miR-133a using transient gene transfection, the transwell assay data showed that miR-133a knockdown resulted in induction of MCF-7 cell migration (*P *< 0.05) and invasion rate (*P *< 0.01) compared with the control cells (Figure 2C). In contrast, ectopic expression of miR-133a in MDA-MB-231 cells resulted in significant reduction of cell migration and invasion (both *P *< 0.01). In addition, the wound healing assay showed that MCF-7 cells with miR-133a knockdown enhanced the scratch wounds closed compared with the controls (*P *< 0.05, Figure 2D).

### FSCN1 is the target gene of miR-133a

**Figure 3:**
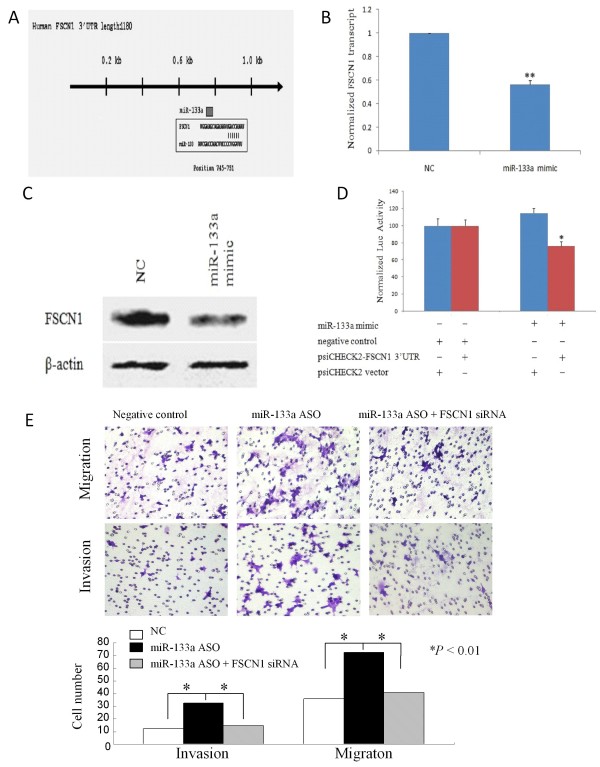
**FSCN1 is the target gene of miR-133a in breast cancer cells**. *A*. Putative conserved target site in the FSCN1 3'UTR was identified with the TargetScan database: The conserved 7-bp seed sequence of miR-133a is aligned to FSCN1 mRNA. *B*. QRT-PCR analysis of FSCN1 mRNA expression. Breast cancer MCF-7 cells were grown and transiently transfected with miR-133a mimic and then subjected to RNA extraction and qRT-PCR analysis. *C*. Western blot analysis of FSCN1 protein expression. MCF-7 cells were grown and transfected with miR-133a mimic or negative control. Total cellular protein was isolated and subjected to Western blot analysis of FSCN1 expression. ß-actin was used as an internal control. *D*. Luciferase reporter assay. MCF-7 cells were transfected with a reporter vector psiCHECK2-FSCN1 3'UTR or psiCHECK2 control vector and miR-133a mimic or negative control. Each transfection was carried out in triplicate. Luciferase assay was performed 48 h after gene transfection. Firefly luciferase activity was normalized to Renilla luciferase activity. *E*. Transwell migration and matrigel invasion assays. MCF-7 cells were grown and transiently transfected with miR-133a ASO, miR-133a ASO plus FSCN1 siRNA, or scrambled sequence oligonucleotides as negative control for 2 days and subjected to migration and invasion assays. Magnification: ×200.

After that, we performed bioinformatical analyses to search for miR-133a targeting genes using the TargetScan [32] database and found that FSCN1 is one of them. Based on the role of FSCN1 in promoting breast cancer progression [22,33,34] and a target gene of miR-133a in esophageal squamous cell cancer and bladder cancer [35,36] as well as identification of one potential binding sites of miR-133a in the FSCN1 3'-UTR (Figure 3A), we first transfected miR-133a mimic into MCF-7 cells and assay FSCN1 mRNA levels in these cells. We found that miR-133a mimic significantly reduced FSCN1 mRNA levels to 56% compared to the controls (*P *< 0.01; Figure 3B). The same is true for FSCN1 protein levels (Figure 3C). Furthermore, luciferase assay demonstrated that miR-133a did bind to FSCN1 3'-UTR site (Figure 3D).

Next, to further explore whether the FSCN1 protein is required for miR-133a-mediated changes in breast cancer cell migration and invasion, we utilized FSCN1 siRNA to knockdown of FSCN1 expression and performed the transwell assays. We found that co-transfection of FSCN1 siRNA and miR-133a ASO significantly abrogated miR-133a ASO-induced MCF-7 cells migration and invasion capacity (Figure 3E).

## Discussion and conclusions

Although tumor invasion and metastasis is the main cause of mortality in patients with solid cancer, our understanding of its molecular and cellular mechanisms is still limited. Discovery of biomarkers to monitor tumor invasion and metastasis for application in clinical practice could to a large extent help clinicians to effectively control tumor metastasis, to determine the risk of recurrence and to predict patient survival. The common methods for miRNA expression analysis were Northern blot, real-time PCR, microarray-based profiling and bead-based technologies in either tissue specimens or blood samples. However, these techniques are inevitably confounded by the heterogeneity of the bio-specimens and thus could not determine the miRNAs expression at the diverse tissue, cell and subcellular origin and level. To this end, we recently utilized *in situ *hybrization approach to screen the miRNAs expression in clinical archival formalin-fixed and paraffin-embedded tissues and found several miRNAs, including miR-133a, was aberrant expressed in breast cancer [10,28]. In the current study, we further confirmed that miR-133a expression was significantly downregulated in breast cancer tissue samples and cell lines, and that loss of miR-133a expression was associated with lymph node metastasis, high clinical stages and shorter RFS of the patients with breast cancer. Moreover, our *in vitro *data demonstrated that miR-133a negatively regulated the invasion and migration potential of breast cancer cells. Taken altogether, our current data indicate that miR-133a may play a role in breast cancer progression and that detection of miR-133a could be further evaluated as a biomarker for prediction of survival of breast cancer.

Recent studies have reported altered expression of miR-133a in several human cancers including esophageal squamous cell carcinoma [35], bladder [36], ileal carcinoid [37] and rhabdomyosarcoma [38]. Ectopic expression of miR-133a significantly inhibited invasion capacity of various human cancer cell lines [35,36]. In breast cancer, miR-133a was found to be reduced by a microarray-based analysis [39]. These previous studies are consistent with our current data, suggesting that miR-133a does play a role as a tumor suppressor gene in regulation of cancer development and progression.

To understand the mechanisms by which miR-133a suppresses tumor cell invasion and metastasis in breast cancer, we identified FSCN1 as one of potentially miR-133a targeting genes using bioinformatic analyses. FSCN1, an actin-binding protein, is essential for actin-based motility-structures formation [19], such as microspikes, lamellipodia and filopodia. FSCN1 is usually not expressed in normal epithelial cells, but is overexpressed in many kinds of tumors [22-27]. Increasing evidence has demonstrated a link between overexpression of FSCN1 and increased tumor cell motility and invasiveness [35,36,40]. In a previous study, overexpression of FSCN1 in nasopharyngeal carcinoma was associated with lymphatic metastasis and knockdown of endogenous FSCN1 expression in nasopharyngeal carcinoma cell lines using siRNA technique greatly reduced cell invasive ability and decreased filopodia formation [40]. Other studies showed that decreased FSCN1 expression through FSCN1 siRNA also weakened invasiveness of esophageal squamous cell carcinoma and bladder cancer cells [35,36]. On the other hand, elevated expression of FSCN1 in breast cancer MDA-MB-435 cell line was greatly enhanced cell dynamics and motility and increased microspike and filopodia formation [34]. These data prompt us to investigate the potential relationship between FSCN1 and miR-133a in breast cancer.

Indeed, we found that breast cancer cells transfected with miR-133a mimics had dramatically lowered levels of FSCN1 mRNA and protein compared with the control cells. The luciferase reporter assay confirmed that FSCN1 is a direct target of miR-133a in breast cancer cells. Moreover, we further demonstrated that FSCN1 was required for miR-133a-mediated changes in breast cancer migration and invasion capacity. Consistent with our data, recent studies in other types of malignances (such as esophageal squamous cell carcinoma and bladder cancer) also showed that miR-133a could directly bind to FSCN1 gene [35,36].

In this study, we confirmed the downregulation of miR-133a in breast cancer tissues and demonstrated association of the lost miR-133a expression with lymph node metastasis, high clinical stages, and shorter survival of these breast cancer patients. We also explored the potential target of miR-133a to be FSCN1 in vitro. However, our current ex vivo data was not supported by any in vivo data, such as a xenograft model to confirm whether restoration of miR-133a expression could reduce breast cancer cell metastasis in vivo. We recognized this limitation but our arguments are i). Such an animal experiment requires stable gene transfection, which may generate selection bias for resistant cell sublines, unless to use an inducible vector; ii). Addition of xenograft assay will not mechanistically answer the question of the role of mir-133a in silence of FSCN1 in breast cancer; and iii). the conclusion of our current ex vivo data is clear and justified, although there is a need to be further verified in different patient populations before use of miR-133a as a biomarker for predication of breast cancer invasion and metastasis in clinic.

## Competing interests

The authors declare that they have no competing interests.

## Authors' contributions

WZS, WCQ, XR, LX, YS and YXQ performed experiments and summarized the data; WZS, ZGH, XXC, ZT and WQ designed experiments; WZS, WCQ and WQ wrote the paper; all authors have read and approved the final manuscript.

## Pre-publication history

The pre-publication history for this paper can be accessed here:

http://www.biomedcentral.com/1471-2407/12/51/prepub
